# The impact of passive ultrasonic irrigation on the bond strength of two different self-etch adhesives to human pulp chamber dentine: a laboratory investigation

**DOI:** 10.1186/s12903-025-05858-x

**Published:** 2025-04-11

**Authors:** Mohammed Turky, Jukka Matinlinna, Monika Lukomska-Szymanska, Venkateshbabu Nagendrababu, Paul M. H. Dummer, Ahmad Abdel Hamid Elheeny, Nermin Alsayed Mahmoud

**Affiliations:** 1 Department of Endodontics, Faculty of Dentistry, Minia University, Minia, Egypt; 2 Department of Endodontics, Faculty of Dentistry, Sphinx University, Assiut, Egypt; 3 Department of Biomaterial Science, Division of Dentistry, Manchester University, Manchester, UK; 4 Department of General Dentistry, Medical University of Lodz, Lodz, Poland; 5 Department of Restorative Dentistry, College of Dental Medicine, University of Sharjah, Sharjah, UAE; 6 School of Dentistry, College of Biomedical and Life Sciences, Cardiff University, Cardiff, UK; 7 Department of Paediatric and Community Dentistry, Faculty of Dentistry, Minia University, Minia, Egypt; 8 Department of Paediatric and Community Dentistry, Faculty of Dentistry, Sphinx University, Assiut, Egypt; 9 Department of Conservative Dentistry, Faculty of Dentistry, Minia University, Minia, Egypt

**Keywords:** Bond strength, Endodontic irrigation, Passive ultrasonic irrigation, Postendodontic restoration, Self-etch adhesives, Syringe irrigation

## Abstract

**Objectives:**

To evaluate the impact of passive ultrasonic irrigation on the micro-tensile bond strength of two different self-etch adhesive systems, including a universal one-step adhesive and a two-step adhesive system, to pulp chamber dentine compared to conventional syringe irrigation.

**Methods:**

Twenty-four extracted human mandibular first molar teeth were chosen according to strict criteria and mounted in epoxy resin blocks. Subsequently, the pulp chambers were exposed using an Isomet cutting machine. The specimens were numbered and assigned to four groups (6 teeth each) based on the canal irrigation method and the adhesive system used as follows: Conventional syringe irrigation in which a universal one-step self-etch adhesive system was applied (CSIU), conventional syringe irrigation in which a two-step self-etch adhesive system was used (CSIT), passive ultrasonic irrigation in which a universal one-step self-etch adhesive system was utilized (PUIU), and passive ultrasonic irrigation in which a two-step self-etch adhesive system was employed (PUIT). Following placement of the final restoration and submission to simultaneous thermo-mechanical cycling (artificial aging) equivalent to 6-month intra-oral aging, the teeth were sectioned and dentine/restoration beams were prepared. The micro-tensile bond strength was evaluated and the failure mode was defined, with a confirmatory evaluation of the dentine-resin interface using a scanning electron microscope. Statistical analysis was conducted using one-way ANOVA and Tukey’s post hoc tests to compare irrigation regimens for each adhesive technique independently, while failure modes of each adhesive system were represented as the frequency for each irrigation method. The significance level was set at 5%, with a confidence interval (CI) of 95%.

**Results:**

The micro-tensile bond strength of composite resin restorations to pulp chamber dentine was reduced significantly with ultrasonic irrigation with more unfavorable failure modes compared to syringe irrigation (*P* < 0.0001), irrespective of the type of adhesive system used. The means of the micro-tensile strength for teeth treated with the two-step adhesive system were 26.1055 ± 4.7611 MPa and 16.0079 ± 3.7665 MPa for CSIT and PUIT, respectively. For teeth treated with the universal adhesive system, the mean for CSIU (20.1818 ± 3.8500 MPa) was significantly higher than that of PUIU (11.2090 ± 2.9928 MPa). The micro-tensile bond strength was significantly greater with the two-step adhesive system compared to the universal adhesive, regardless of the irrigation method (*p* < 0.05). An adhesive layer with varying thickness was noted in all groups, displaying distinct morphological features.

**Conclusions:**

Within the limitations of the present laboratory investigation, ultrasonic irrigation may negatively affect the bond between composite resin restorations and pulp chamber dentine compared to conventional syringe irrigation. The two-step self-etch adhesive tended to achieve a stronger bond to pulp chamber dentine than the universal one-step self-etch adhesive.

**Clinical relevance:**

While ultrasonic irrigation would be essential for effective root canal debridement and disinfection, it is imperative for clinicians to consider its potential adverse effects. This method may considerably impact the bond strength of composite resin restorations to the pulp chamber dentine, particularly when compared to conventional syringe irrigation. In root canal-treated teeth, a two-step self-etch adhesive system might be more effective in maximizing the bond strength to pulp chamber dentine than a universal adhesive system. However, these findings were concluded under the conditions of the present study and must be interpreted cautiously. Further research is recommended to validate these results and fully understand the clinical ramifications of ultrasonic irrigation on adhesive performance in different dental situations.

**Clinical trial number:**

Non-applicable. Conducting the current experiment was limited to the approval of the local Research Ethics Committee at the Faculty of Dentistry, Minia University, Egypt (Committee No. 106, Registration No. 910, Date: April 30, 2024).

**Supplementary Information:**

The online version contains supplementary material available at 10.1186/s12903-025-05858-x.

## Introduction

Endodontic instruments used for root canal preparation have evolved substantially in recent times [1]. However, even with these improvements, substantial portions of the root canal system remain untouched by instruments [2]. These non-instrumented areas typically harbour pulp remnants, dentine debris, as well as residual microbial biofilms [3, 4]. It has been established that these unprepared areas can negatively impact treatment outcomes [5]. Consequently, root canal irrigation plays an indispensable role in root canal debridement and disinfection, particularly for the areas beyond the reach of mechanical instruments [6].

Conventional syringe and needle (CSI) is the most popular method for delivering irrigants inside the root canal system [6]. CSI relies on the positive pressure of the fluid through the needle and the viscosity of the irrigant to achieve flow throughout the root canal system [7]. However, the maximum effect of CSI has been reported to be confined within 1 mm of the needle tip, leaving areas beyond this distance at risk of being inadequately cleaned and disinfected which may adversely affect the outcome of treatment [8]. In addition, since the root canal system is closed-ended, air bubbles are likely to accumulate apically, leading to a phenomenon termed “apical vapour lock” [9], which can impede the deep penetration of irrigating solutions into the important apical part of the root canal. This region is the most critical section to debride as remaining microorganisms may cause persistent apical periodontitis [9, 10]. The viscosity and thus diminished flow characteristics of the commonly used root canal irrigants, sodium hypochlorite (NaOCl) and ethylenediaminetetracetic acid (EDTA), may also impact the delivery of irrigants to this critical apical region. In order to address these issues, approaches to improve the flow and effectiveness of endodontic irrigants and overcome the apical vapour lock phenomenon have been developed. One of these approaches involves the agitation of the irrigating solutions [11, 12].

Agitation of root canal irrigants uses mechanical, physical, or other forms of energy to enhance their flow into the complex root canal system [7]. Despite the diverse range of techniques for irrigant agitation, passive ultrasonic irrigation (PUI) remains the most widely used [6]. It has been reported that ultrasonic agitation creates acoustic streaming and cavitation generating energy within the solutions, which exerts greater shear stresses against the canal walls [13, 14]. This energy can disrupt the attached pulp remnants, debris, and/or residual bacterial biofilms, along with the elimination of the apical vapour lock [13–15]. Additionally, it has been suggested that ultrasonic activation raises the temperature of irrigants, which reduces their viscosity and hence increases their flow within the canal system [16] and enhances their chemical reactivity thereby improving their antimicrobial and tissue-dissolving capabilities [16–18]. The distinctive role played by ultrasonic agitation in root canal debridement has been reported [6, 19], and a recent systematic review and meta-analysis of randomized clinical trials disclosed its superiority over conventional syringe irrigation regarding root canal disinfection [15].

Along with thorough root canal preparation and filling, the quality of the coronal restoration has been reported to have an impact on the outcome of root canal treatment and retreatment [20–22]. Indeed, numerous studies have highlighted the importance of coronal restoration in preventing root canal re-infection and maintaining periapical health [23–26]. It has been reported that teeth with adequate coronal restorations with no evidence of marginal discrepancy, recurrent caries, or discoloration at the restoration margin have three times better periapical healing rates compared to those with inadequate restorations [27, 28].

Bonded restorations such as composite resin restorations, are one of the most effective treatment options for preventing coronal microleakage and ensuring a proper coronal seal [29]. When using composite resin, establishing a strong bond between the material and coronal dentine is therefore of utmost importance [22]. It has been reported that coronal dentine can be affected by root canal irrigation [30, 31]. A substantial body of literature has shown conflicting outcomes concerning the impact of root canal irrigants, in particular NaOCl, using CSI on the bond strength to coronal dentine [30–33].

Notwithstanding the benefits of PUI during root canal irrigation, the impact of ultrasonic agitation on the bond strength of composite resin restorations has not been explored. Therefore, the present laboratory study sought to evaluate the influence of PUI on the bond strength of composite resin restorations to dentine. Two types of adhesives were used to address recent research that revealed that the effect of NaOCl varied according to the type of adhesive [34]. Dental adhesive technology has witnessed remarkable advancements with the introduction of self-etch adhesives. These adhesive systems were evolved to reduce technique sensitivity and streamline clinical procedures. Self-etch adhesives are categorized as one-step and two-step self-etch adhesive systems. One-step self-etch adhesive systems are presented as all-in-one systems, incorporating etching, priming, and bonding into a single step. These adhesive systems encompass a mixture of hydrophobic and hydrophilic monomers, acidic functional monomers, organic solvents, and water. Although these adhesive systems have certain advantages such as shorter chair time and fewer clinical procedures, reducing post-operative sensitivity, their hydrophilic nature can negatively impact bond longevity due to fluid movement through the adhesive layer. On the other hand, the two-step self-etch adhesive systems consist of distinct etching primers and bonding agents. While the etching primer simultaneously etches and primes the tooth structure by the acidic monomer, the bonding agent seals the dentine following solvent evaporation. Compared to the one-step adhesives, this two-step adhesive system provides superior control over the etching and priming procedures and better bond stability. Despite these differences, both self-etch adhesives share similar features such as reduced technique sensitivity and ease of application, making them frequently utilized in clinical practice, particularly since the available evidence does not conclusively favor one system over the other [35].

Bonding to pulp chamber dentine is crucial to enhance retention, and optimize the coronal seal [34]. When assessing the effectiveness of the bond strength of dental adhesives, particularly in intricate environments like the pulp chamber, utilizing a precise and reliable evaluation method is essential. Among various techniques available, the micro-tensile bond strength test offers detailed insights into the performance of the adhesive for several reasons. Firstly, this method facilitates the even distribution of stresses along the bonding area, minimizing the risk of cohesive failures and providing a meticulous evaluation of the adhesive effectiveness. Secondly, this test allows for using multiple specimens from a single tooth, reducing the number of teeth needed for a study. Thirdly, this technique primarily results in adhesive failure, offering a clearer understanding of the interaction between dentine and adhesive. Lastly, it boasts a superior discriminative ability to assess different adhesives and substrates. This ability is important for clinicians seeking to select the most appropriate adhesive materials for specific applications. Taking into account all these merits, the micro-tensile bond strength test emerges as the most appropriate method to assess the robustness of the bond of different adhesive systems to the pulp chamber dentine [36]. Therefore, the present investigation aimed to assess the impact of PUI on the micro-tensile bond strength of two distinct self-etch adhesive systems, including the universal one-step adhesive and a two-step adhesive system, to the pulp chamber dentine compared to CSI.

The null hypothesis tested was that there is no significant difference between PUI and CSI regarding the micro-tensile bond strength of the two different self-etch adhesive systems to the pulp chamber dentine.

## Materials and methods

The manuscript has been written according to the Preferred Reporting Items of Laboratory studies in Endodontology (PRILE 2021) guidelines [37]. A summary of the study is provided in the PRILE 2021 flowchart (Supplementary Fig. 1). 

### Ethical approval

The current laboratory investigation was conducted on extracted human teeth after the approval of the local Ethics Committee at the Faculty of Dentistry, Minia University, Minia, Egypt with registration No. 910. The study adhered strictly to the principles outlined in the Helsinki Declaration as well as to all pertinent guidelines and regulations governing research involving human subjects. Prior to the collection of teeth, informed written consent was obtained from each participant, ensuring that all donors were fully aware of the study’s purpose, procedures, and potential risks. This commitment to ethical standards underscores the importance of preserving the rights and welfare of participants throughout the research process.

### Sample size estimation

In order to achieve statistical significance between the assumed difference in tensile stress at maximum (MPa) in the PUI group and CSI group (14.6 with a standard deviation for beams of the 4 groups at 6.6, based on the findings of the pilot study), a minimal sample size of 14 beams in each group was required. This was accomplished by setting the power at 0.80 and α at 0.05 using PASS 11th release (Hintze J. (2011): PASS 11. NCSS, LLC. Kaysville, UT, USA). To ensure the certainty of the assumption, the estimated minimal sample size of 14 beams per group was increased to 15 beams.

### Teeth selection

A total of forty teeth were initially screened from which dimensionally and anatomically matched twenty-four recently extracted human sound mandibular first molars with fully formed two roots were selected. Teeth were collected from healthy patients categorized as class I ASA (American Society of Anesthesiologists) and aged between 20 and 30 years old in the outpatient clinic at the Faculty of Dentistry, Minia University, Minia, Egypt. Extractions of teeth, which were due to periodontal reasons, were performed with minimal trauma. To ensure proper anatomical matching in tooth dimensions, morphology, pulp space volume, and morphology, three-dimensional imaging software (Papaya 3D Plus, Genoray, Gyeonggi-do, Korea) was utilized. This detailed imaging process was essential to uniform the anatomical variables that could influence the outcomes of root canal treatment, specifically focusing on the bond strength to dentine of the pulp chamber. The selected teeth had equivalent angles of root and/or root canal curvatures, which were determined using Schneider’s method [38], radii, and identical root canal configurations with a Vertucci’s type IV [39] in the mesial root and Vertucci’s type I in the distal one. Teeth with caries, root canal calcifications, previous restorations or root canal fillings, and internal or external root resorptive lesions were excluded and replaced with teeth that met the inclusion criteria. Furthermore, teeth were examined for evidence of cracks and/or fractures under a magnification of 20X using a dental operating microscope (DOM) (Magna Labomed, Labo America, CA, USA). The teeth underwent ultrasonic cleaning, placed in 2.5% NaOCl (El Nasr Company for Intermediate Chemicals, Giza, Egypt) for 30 min, and then stored at 4 °C in a 0.1% thymol solution (Formula e Acao, São Paulo, SP, Brazil) until testing for a maximum one month following extraction [40].

### Mounting and preparation of specimens

The tooth roots were embedded in epoxy resin blocks (Kemapoxy, Cairo, Egypt) with the resin level positioned 2 mm apical to the cementoenamel junction (CEJ) using a hollow metallic cylindrical template. To stabilize the mould during specimen preparation, an external copper ring (30 mm in diameter and 25 mm in height) encircled the internal split Teflon part, which had a 20 mm diameter and 20 mm height.

Teeth were sectioned at the level of the pulp chamber roof using an Isomet 4000 cutting machine (Buehler, Lake Bluff, IL, USA) with a diamond disc (Buehler Diamond Wafering Blade 11-4245) running at 200 rpm submerged in water. The pulp chamber cavity and the surrounding dentine thickness were standardized using a periodontal probe [41]. Moreover, all teeth were adjusted to a length of 17 mm.

### Experimental groups

The sample distribution utilized stratification, with specimens matched in pairs according to their dimensions and anatomical features. Teeth that shared similar dimensions and anatomical characteristics were categorized into four distinct groups. In other words, each set of six anatomically and dimensionally matched teeth was assigned separately to mitigate the risk of bias from anatomical variations among the selected samples.

The prepared teeth were numbered and divided into four equal groups (6 teeth each; 5 teeth for testing the micro-tensile bond strength and 1 tooth for scanning electron microscope evaluation) according to the irrigation protocols and the type of adhesives as follows:

#### CSIU group

In this group, the pulp chamber walls were refined using a tapered carbide fissure bur (Komet H33L, Komet Den Komet Dental, Braseler GmbH & Co. KG, Lemgo, Germany) mounted on a high-speed handpiece with water-coolant. Subsequently, the pulp chamber was soaked in 2.5% NaOCl for 1 min prior to root canal preparation. Root canal preparation was conducted in compliance with the recent guidelines of root canal cleaning and shaping [42]. Root canal patency was checked using a stainless-steel K-file size 10 (Dentsply Maillefer, Ballaigues, Switzerland). The working length was determined by inserting a stainless-steel size 10 K-file until it was visible at the apical foramen, then subtracting 1 mm from this measurement. All root canals were standardized to have initial apical sizes equivalent to ISO size 15 for mesial root canals and ISO size 25 for the distal canals. Afterward, root canals were instrumented up to an apical size of 35, 0.04 taper in the mesial canals, and up to size 40, 0.04 taper in the distal canals using a full-sequence rotary system (Hyflex CM; Coltene Whaledent, Cuyahoga Falls, OH, USA) adapted on an electrical controlled torque endodontic motor (TriAuto mini; J. Morita MFG, CORP, Tokyo, Japan). The speed and torque settings were adjusted per the manufacturer’s recommendations. Between files, root canals were irrigated with 2 ml of 2.5% NaOCl for 20 s delivered through a 30-gauge side-vented closed-end irrigating needle mounted on a plastic syringe (Fanta Dental Materials, Shanghai, China). A final irrigation protocol including 10 mL of 2.5% NaOCl for 2 min followed by 10 mL of 17% EDTA for 2 min, with an intermediate irrigation of saline solution with the same volumes and for the same duration was accomplished. Finally, a final flush of saline solution was conducted, followed by dryness of the root canals utilizing matched paper points (Coltene Whaledent, Cuyahoga Falls, OH, USA).

Immediately following the completion of root canal preparation, adhesives were applied to the pulp chamber dentine. Prior to adhesive application, the entire pulp chamber of each tooth was cleaned with water and gently air-dried. To ensure a surface conductive to bonding, the air-abrasion method utilizing silica-coated alumina particles was employed to establish a rough surface, enhancing the mechanical interlocking between the adhesive and dentine. Additionally, it is capable of eliminating the residual debris while facilitating chemical bonding through the silica coating. Subsequently, the sandblasted surfaces were rinsed thoroughly with water, to remove the residual debris generated during the sandblasting method, and air-dried. The universal adhesive system (one-step self-etch adhesive) (Bisco, Schaumburg, IL, USA) was applied to the dentinal walls of the pulp chamber per the manufacturer’s instructions, a single coat was applied and agitated for 20 s using a micro brush (3 M ESPE, St. Paul, MN, USA), a gentle air drying was used to remove the excess solvent then light cured for 20 s by light-emitting diode curing unit (Blue phase, Ivoclar Vivadent, Zurich, Switzerland), with a light intensity of 1200 MW/cm² and wavelength bandwidth of 400–515 nm. The curing tip was directed in intimate contact with tooth/restoration surfaces. The Aphrodite LED radiometer CM-2500 (Motion Medical Supplies & Equipment Corporation, Sanzhong, Taiwan) was employed to measure and standardize the light output.

#### CSIT group

In this group, the root canal procedures were completed in the same manner as in the CSIU group. In contrast, restorative procedures were different. The composite resin was bonded using the two-step self-etch adhesive system (Kurary Noritake Dental, Okayama, Japan) per the manufacturer’s instructions. The primer was initially applied for 20 s, after which oil-free air was gently blown for 5 s. Then, the bonding agent was applied, rubbed for 20 s, gently blown to ensure uniform thickness, and light-cured for 10 s.

#### PUIU group

In this group, the root canal and restorative procedures were repeated as previously mentioned in the CSIU group. Moreover, Passive intermittent ultrasonic agitation of an additional 2 ml of 2.5% NaOCl in three successive cycles of 20 s each was achieved following root canal preparation as a part of the final irrigation protocol prior to EDTA irrigation. This process was conducted using an E1 Irrisonic tip (Helse Dental Technology, São Paulo, SP, Brazil) corresponding to size 20, 0.01 taper with a power setting of 20%. The tip was passively inserted up to 3 mm short of the working length.

#### PUIT group

In this group, root canal procedures and passive ultrasonic irrigation were carried out as described before in the PUIU group, While the restorative procedures were performed as in the CSIT group.

### Tooth restoration

The pulp chambers were filled with a final composite resin restoration (Tetric N ceram, Ivoclar Vivadent, Zurich, Switzerland) applied using a gold-plated composite resin applicator (American Eagle Composite Set, Heidelberg, Germany). The restoration was applied incrementally in 2 mm layers, with each layer being light-cured for 20 s, using the same light characteristics mentioned previously, until the surface layer was reached.

To achieve a high degree of homogeneity across the different study groups and ensure that the experimental procedures were aligned with the best practice, thereby reducing the variability of the study results, all root canal procedures were conducted by a single specialist with 21 years of experience in endodontics (M.T.), while the subsequent restorative procedures were performed by an experienced operator with 24 years of practice in conservative dentistry (N.A.M.).

The materials used in the study, their specifications, composition, manufacturer, and LOT No. are listed in Table 1.

**Table 1:** The specification, composition, manufacturer, and LOT no. of the materials used in the study

Material	Specification	Composition	Manufacturer	LOT Number
All-bond universal dental adhesive	One step self-etch adhesive	10-methacryloyloxydecyl dihydrogen phosphate (MDP), Bisphenol A di glycidyl methacrylate (Bis-GMA), 2-hydroxyethyl methacrylate (HEMA), Ethanol, Water, Initiators	Bisco, Schaumburg, Illinois, USA	1,900,004,492
Clearfill™ SE bond	Two-step self-etch adhesive system	1) **PRIMER**: 10-Methacryloyloxydecyl dihydrogen phosphate (MDP), 2-Hydroxyethyl methacrylate(HEMA), Hydrophilic aliphatic di-methacrylate, dl-Camphorquinone, Water. 2) **BOND**: 10-Methacryloyloxydecyl dihydrogen phosphate (MDP), 2-Hydroxyethyl methacrylate (HEMA), Bisphenol A di-glycidyl-methacrylate (Bis-GMA), Hydrophobic aliphatic di-methacrylate, dl Camphorquinone, Initiators, Accelerators, Silanated colloidal silica	Kurary Noritake Dental, Okayama, Japan	000201
Tetric N Ceram	Nano-hybrid resin composite	**Resin matrix**: Dimethacrylates (1920% weight) **Inorganic fillers**: · 80–81% weight or 55–57%volume · Barium glass, ytterbium trifluoride, mixed oxides and copolymers · 0.4–0.7 micron · Nano-hybrid ·	Ivoclar Vivadent, Zurich, Switzerland	Z04Hcj

To avoid over-drying of dentine, all the root canal and restorative procedures for all groups were accomplished in a single session. Subsequently, teeth were kept in distilled water for 24 h to ensure complete polymerization, prior to submission to thermo-mechanical fatigue, thus simulating clinical conditions.

### Thermo-mechanical fatigue

A four-station multi-modal ROBOTA chewing simulator (ACTA Fatigue Tester, Amsterdam, Netherlands) was utilized for mechanical aging, featuring servomotor control (Model Ach-09075dc-T, Ad-Tech Technology, Berlin, Germany) and a thermo-cyclic protocol. The ROBOTA simulator enabled simultaneous movements in both horizontal and vertical directions under thermodynamic conditions.

Each chamber consists of an upper hardened steel stylus holder that can be securely fastened to serve as an antagonist, and a lower plastic sample holder for placing the specimen. A weight of five kilograms, equivalent to a chewing force of 49 N, was applied during the tests.

The following parameters were set for the chewing simulation:

- A cycle frequency of 1.6 Hz,
- A rising/forward speed of 90 mm/s,
- A descending/backward speed of 40 mm/s,
- A horizontal movement of 1 mm, and a vertical rise of 3 mm.

The specimens were submitted to a total of 75,000 cycles, including 6,000 thermal cycles (ranging from 5˚C to 55˚C, with a dwell period of 25 s) to mimic intraoral aging of 6 months [43].

### Tooth sectioning and beam Preparation

**Fig. 1:**
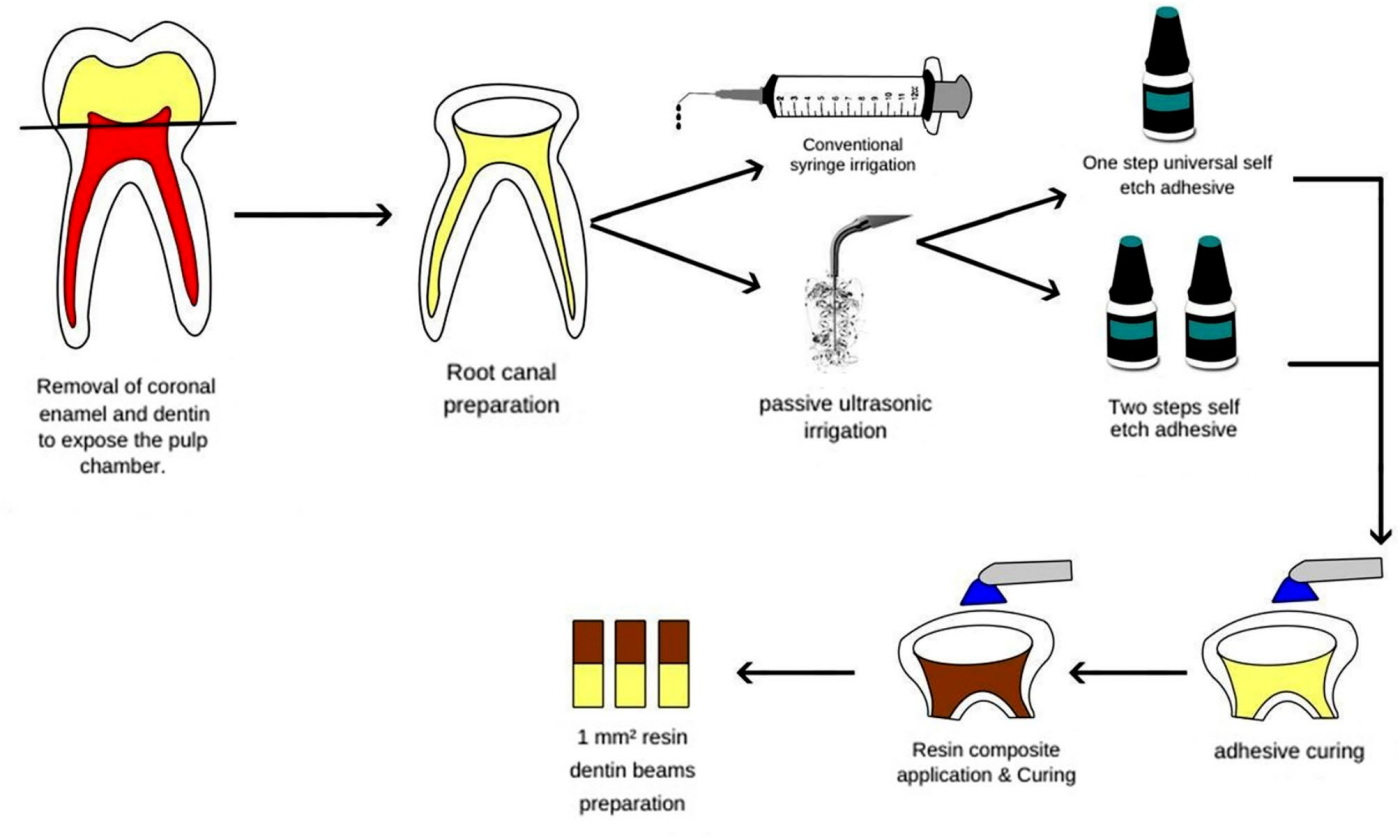
Schematic diagram summarizing the methodology

The metal housing of the Isomet cutting machine was secured with two screws to firmly hold resin blocks soldered at the square base, ensuring uniform 90° cutting in both buccolingual and mesiodistal directions when mounted into the diamond saw machine. After being mounted in the gripping attachment, the restored teeth were sequentially sectioned at 2050 RPM under generous coolant, using a 0.3 mm thick diamond-coated disc (Buehler, Lake Bluff, IL, USA). Serial sectioning was done in the buccolingual direction, followed by a 90° clockwise rotation and a mesiodistal section, then a final horizontal cut was made at the cementoenamel connection level with a cross-section area of 1.0 ± 0.1 mm², as measured by a digital caliper. The beams were prepared from the axial buccal wall of the pulp chamber. After excluding any invalid beams, 15 from 5 teeth were selected for each group to be tested for bond strength (Fig. 1).

### Measurement of micro-tensile bond strength

Acrylate-based glue (Akfix 705 fast adhesive, İstanbul, Turkey) and a glue accelerator were used to attach each beam to the jig. Each beam was positioned at least 1 mm away from the adhesive interface. Subsequently, the jig was inserted into the 500 N load cell universal testing apparatus (Instron, Norwood, MA, USA) for further testing. A tensile stress was applied at a cross-head speed of 0.5 mm/min until the specimen’s bonding failed. Using Bluehill Lite software (Instron, Norwood, MA, USA), the bond strength was estimated in MegaPascal (MPa).

### Failure mode evaluation

In order to identify the mode of failure, each fractured beam was inspected under a 30X magnification MA 100 Nikon stereomicroscope (Nikon, Tokyo, Japan). Failure modes were classified as adhesive (at the interface of tooth-composite resin), cohesive (failure inside the composite resin), and mixed failure (when more than 25% of the failure was adhesive) [44].

### Scanning electron microscope evaluation of dentine-resin interface

Two slices of the lateral wall pulp chamber dentine-resin interface were obtained from the specimens (one tooth per study group) after being sectioned perpendicularly for 24 h using a low-speed diamond bur. The slices were embedded using Epoxy (Kemapoxy, Cairo, Egypt) and allowed to cure for 8 h at room temperature. Subsequently, under running water, the slices were polished using SiC papers (#600, #800, and #1000) in order, and then diamond pastes (6, 3, and 1 mm). After polishing, the slices were cleaned in ultrasonic equipment using distilled water. Next, they were exposed to 1 mol/L hydrochloric acid for 30 s, 5.25% NaOCl for 5 min, and distilled water for rinsing [34]. After 24 h of drying at room temperature, all of the slices were sputtered with gold for 120 s, and SEM (JEOL JSM 6510 lv, Tokyo, Japan) observation was performed at a magnification power of 2000x [45].

The evaluation process was conducted by an independent examiner who was blinded to the experimental procedures.

### Statistical analysis

Data were tested for the assumption of normality and variance homogeneity using the Kolmogorov-Smirnov and Shapiro-Wilk tests. To compare different independent groups, one-way ANOVA and Tukey’s post hoc tests were used to assess the significance of differences between pairs of group means (i.e., comparing irrigation techniques for each adhesive). The data of failure modes of each adhesive system were represented as the frequency for each irrigation technique. The significance level was set at 5% and a 95% confidence interval (CI).

## Results

### Micro-tensile bond strength

When the four groups were compared, the highest mean micro-tensile strength values at maximum load were found in conventionally irrigated specimens restored with the two-step adhesive system. The difference between the four groups was statistically significant (*p* < 0.05).

The difference between the two irrigation techniques was statistically significant regardless of the type of adhesive system (*p* < 0.0001). For teeth treated with the two-step adhesive system, the means of CSIT and PUIT were 26.1055 ± 4.7611 MPa with a 95% CI of 23.4689 to 28.74215 and 16.0079 ± 3.7665 MPa with a 95% CI of 13.9221 to 18.0937. In specimens treated with the universal adhesive system, the mean of micro-tensile strength at the maximum load in the CSIU group was 20.1818 ± 3.8500 MPa with a 95% CI of 18.0485 to 22.3127, which was significantly higher than in the PUIU group with a mean of 11.2090 ± 2.9928 MPa with a 95% CI of 9.5517 to 12.8663.

**Fig. 2:**
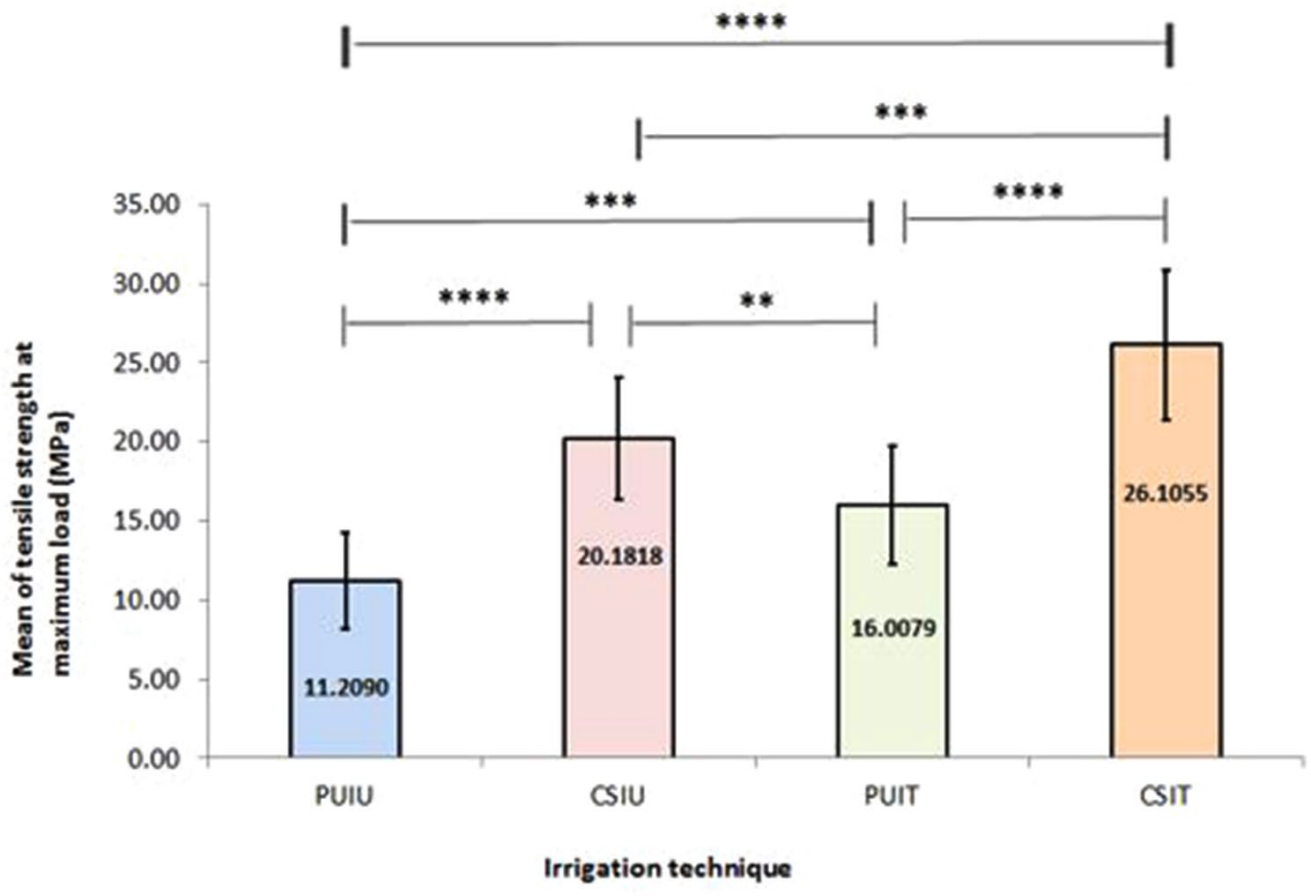
Bar chart showing the effect of irrigation technique and adhesive system on micro-tensile bond strength of composite resin restorations to pulp chamber dentine

A paired comparison revealed that the means of the maximum micro-tensile strength values with the two-step adhesive system were significantly higher than those restored with the universal adhesive system either with the CSI method (*p* = 0.001) or with PUI (*p* = 0.007) (Table 2; Fig. 2).

**Table 2:** Effect of irrigation technique on the mean micro-tensile bond strength values (MPa) of the experimental groups

Irrigation technique	Mean ± SD	95% CI	
		Lower limit	Upper limit
PUIU	11.2090 ± 2.9928	9.5517	12.8663
CSIU	20.1818 ± 3.8500	18.0485	22.3127
PUIT	16.0079 ± 3.7665	13.9221	18.0937
CSIT	26.1055 ± 4.7611	23.4689	28.74215

PUIU: passive ultrasonic irrigation in which a universal adhesive system was utilized, CSIU: conventional syringe irrigation in which a universal adhesive system was applied, PUIT: passive ultrasonic irrigation in which a two-step adhesive system was employed, and CSIT: conventional syringe irrigation in which a two-step adhesive system was used

### Failure mode

Adhesive failure was the dominant failure mode in the four groups with the highest percentage in the PUIU group (67%) followed by the PUIT group (60%), CSIT group (53%), and CSIU group (47%). The cohesive failure percentage was the lowest among all tested groups with a similar value of 13%. Mixed failure mode was the highest among teeth irrigated conventionally with a percentage of 47% in the CSIT group and 40% in the CSIU group. For teeth irrigated with the passive ultrasonic approach, the mixed failure mode percentages in the PUIT and PUIU groups were 27% and 33%, respectively.

### Scanning electron microscope evaluation of dentine-resin interface

**Fig. 3:**
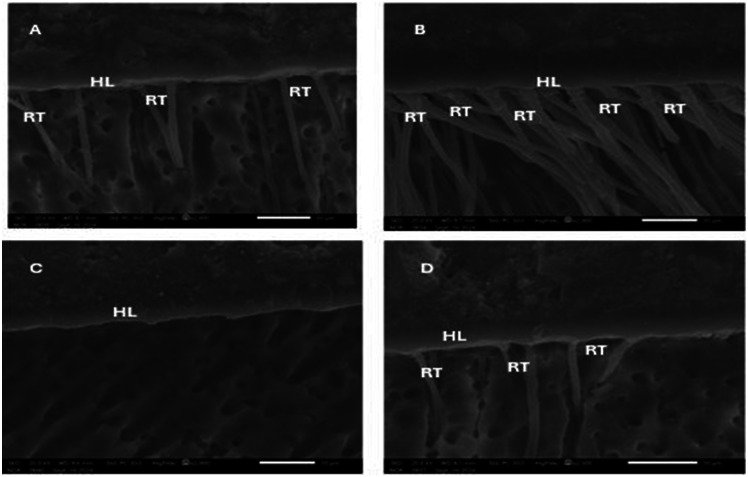
SEM images of dentine/resin interfaces from the experimental groups; **A** CSIU group, **B** CSIT group, **C** PUIU group, and **D** PUIT. HL: hybrid layer and RT: resin tags

SEM images for the groups revealed the presence of a thick hybrid layer with multiple long funnel-shaped resin tags in the CSIT group, while in the CSIU group, a thinner hybrid layer was noted compared to that of the CSIT group with fewer resin tags. In the PUIU group, the hybrid layer was slightly thicker than in the CSIU group with a complete absence of resin tags, but few short resin tags with unequal distribution were observed in the PUIT group (Fig. 3).

## Discussion

Despite the predictable outcomes associated with chemo-mechanical canal preparation including mechanical instruments and conventional syringe irrigation, additional improvements in root canal cleaning and disinfection can be achieved through irrigation agitation [6, 15, 19]. It is worth noting that the outcome of root canal treatment depends not only on adequate root canal debridement, disinfection, and filling but also on the quality of the coronal restoration [20–22]. Undoubtedly, this triad ought to interact seamlessly to complement each other. Apart from their role in restoring function, anatomy, and aesthetics, coronal restorations, especially bonded restorations, are crucial for creating a proper seal [23–26, 46]. Bonding between dentine and resin occurs when the resin penetrates the treated dentinal surface, creating micromechanical interlocking with the dentine collagen and forming a hybrid layer [47]. During root canal treatment, irrigation solutions can affect the bond between the pulp chamber dentine and adhesive material [48]. While some studies have investigated the effect of different irrigation solutions and techniques on the quality of root canal filling [49, 50], others have evaluated the influence of various irrigants on the efficiency of the bond strength of the coronal restoration [30–33]. So far, the influence of irrigation agitation on the bond strength of final restorations has not been evaluated. Considering passive ultrasonic activation is the most popular method for agitation of root canal irrigation, the current study aimed to assess the influence of passive ultrasonic irrigation compared to conventional syringe irrigation on the micro-tensile bond strength of two different adhesive systems to pulp chamber dentine.

It has been emphasized that composite resin restorations can internally reinforce root canal-treated teeth with subsequent increased load capacity of the remaining tooth structure [51–53]. This largely depends on the adhesive system employed through micromechanical interlocking with a hybrid layer. It has also been observed that the robustness of bond strength to coronal dentine can vary due to NaOCl irrigation according to the type of adhesive [34]. Therefore, two adhesive systems were used to consolidate the outcomes of the current study. Indeed, the present study corroborated prior research [54–56] and showed that the two-step adhesive system significantly outperformed the universal one-step adhesive system, irrespective of the irrigation method. The superior performance of the two-step adhesive was probably due to the second step which provides a hydrophobic cover over a hydrophilic primer, hampering water intake and subsequent bond degradation. Furthermore, it might offer an additional layer that acts as a stress-absorbing zone to the adhesive interface during polymerization-induced composite shrinkage [57].

Although some reports have concluded that passive ultrasonic improves the bond strength of the root canal filling to radicular dentine compared to conventional syringe irrigation [50, 58], others found no discernable differences in the bond strength to root dentine between the two irrigation protocols [59, 60]. The present findings highlight that ultrasonic irrigation significantly compromised the micro-tensile bond strength to pulp chamber dentine with more unfavorable failure modes, regardless of the type of the adhesive system, compared to syringe irrigation. Therefore, the tested hypothesis was rejected. Considering prior reports that have shown the negative impact of NaOCl delivered through the conventional syringe method on the bond strength to coronal dentine due to the released oxygen by-product that can adversely affect the adhesive system polymerization along with the NaOCl-induced damage to the organic matrix [30, 31], the current findings might be attributed to the expected increased flow and action of NaOCl due to the high energy generated within the solution during ultrasonic activation that can force the irrigant to more distant areas than the conventional syringe technique [13, 14]. Furthermore, as previously mentioned, ultrasonic agitation can warm NaOCl, which reduces its viscosity and increases its flow [16]. In addition, this warming is able to increase the chemical reactivity of NaOCl [16–18].

In an important effort to enhance the translation of the present work outcomes into a trustworthy clinical setting, the current investigation has considered all confounding factors overlooked in most laboratory investigations such as sample selection based on strict criteria, extraction time and technique, specimen age, storage conditions, and thermo-mechanical cycling.

Due to their early eruption, mandibular first molars are the most frequent tooth type affected by carious lesions and often require root canal treatment [61]. Therefore, mandibular first molars extracted with minimal trauma were selected in the present study. One of the most critical aspects of research design is establishing a reliable baseline because anatomical variations among the samples can impact the results. To overcome this issue, the selected teeth in the present investigation were carefully chosen and allocated to the different groups based on pair-matching using 3D imaging. The criteria for selection included teeth with an equal number of root canals, identical canal configurations, and comparable tooth dimensions and root canal volumes. By standardizing these features, the current investigation attempted to minimize variability during root canal procedures, including instrumentation and irrigation processes, and the subsequent bonding to the pulp chamber dentine. In addition, to guarantee consistent conditions for adhesive bonding, teeth that had comparable pulp chamber volumes were chosen. This attention to anatomical details during sample selection was crucial in creating a precisely controlled environment, allowing for drawing more valid and reliable conclusions from the current research.

Tooth age is often neglected in many ex vivo studies. Dentine is a hard tissue structure that constitutes 60% inorganic part (apatite crystal) which is responsible for the compressive strength and hardness, 30% organic part (collagen network) which provides elasticity, and 10% water [62, 63]. Any change in these components may contribute to modifications in the physical properties of dentine. Over time, dentine undergoes physiological changes, including a reduction in peritubular fluid, narrowing of the tubular diameter, and calcification [62]. Previous reports have highlighted that dentine from young patients (17–30 years old) has greater fatigue strength compared to dentine from older patients (50–80 years old) [64]. Furthermore, it has been reported that bond strength to dentine could be affected by tooth age [65]. As a result, this experiment was conducted using intact teeth collected from healthy adult patients of a comparable age range (20–30 years old) in an attempt to ensure consistency in dentine characteristics and minimize the risk of any pathological or physiological changes.

Storage conditions in terms of time and storage media are also one of the pertinent issues that can impact dentine properties and subsequent bond strength [66–68]. As per technical specification 11–405, in the field of dental materials, bond strengths should preferably be evaluated immediately following extraction, but this is often not feasible. The most significant alterations seem to occur in the first days or weeks post-extraction. Consequently, teeth should be tested 1 month after extraction, but not more than 6 months thereafter. Therefore, all teeth in the current study were freshly extracted and stored in the same storage media for a period not exceeding 1 month before testing. Given this short-term storage in the present experiment, the thymol solution proved to be a viable option for preserving the recently extracted teeth without compromising the dentine’s mechanical characteristics and hence the bond strength [69].

Thermo-mechanical fatigue can replicate the natural stresses submitted to the teeth over time during oral function that may cause collagen degradation and adversely impact the bond strength of the coronal restoration [70–72]. Thus, thermo-mechanical cycling was considered in the present study to mimic the clinical scenario.

There is a lack of a well-established consensus on the concentration of NaOCl when used as an endodontic irrigant. Although the reported tendency towards using full-strength NaOCl for more effective tissue dissolution and root canal disinfection [73, 74], it may irritate the periapical tissues and negatively affect the mechanical and structural properties of dentine [75, 76]. Moreover, no discernible differences in treatment outcomes between the different concentrations of NaOCl have been reported [77]. Therefore, a concentration of 2.5% was used in the present experiment and verified using iodometric titration in order to ensure uniform concentrations.

Despite its undeniable antimicrobial effectiveness, unique organic tissue dissolving capacity, and being a lubricant for mechanical instruments during root canal preparation, NaOCl cannot be the only root canal irrigant due to its inability to dissolve the inorganic component of the smear layer [74, 78]. Given this major limitation, NaOCl should be supplemented with a chelating agent such as 17% EDTA to enhance smear layer removal [74, 78]. Not only does EDTA improve the removal of the smear layer, but it can also boost the antibiofilm action of NaOCl, despite its minor antimicrobial capabilities, through the detachment of the biofilm structures from the canal wall [79, 80]. Consequently, while NaOCl is typically used between mechanical files, it should be adjunct with EDTA as a final rinse at the end of chemo-mechanical preparation. Taking into account the disastrous consequences of combining both irrigants [74, 78] as well as the potentially detrimental impact on dentine structure that might result from the use of EDTA preceding NaOCl [81], the standard protocol including the alternate use of 10 mL of 2.5% NaOCl followed by 10 mL of 17% EDTA, each for 2 min [78], with an intermediate rinse of saline solution employed in the present investigation. To avoid the unremitting EDTA-induced softening of the canal walls, a final flush of saline solution was used [74].

Research has shown that the effects of NaOCl and EDTA on coronal dentine rely on both the duration of exposure and the concentration used [82]. Given that, in this investigation, the time and concentrations of the irrigation solutions were precisely standardized across all samples.

Intermittent or continuous passive ultrasonic agitation are two forms of ultrasonic irrigation. Repetitive oscillation can produce intense streaming, enhancing cleaning effectiveness and resulting in more efficient biofilm removal than continuous activation for the same period [83, 84]. Furthermore, the regular replacement of the irrigating solutions makes up for the loss of irrigant out of the pulp chamber and its consumption in chemical reactions [85]. For these reasons, intermittent ultrasonic activation was chosen in the present experiment. To maximize the effects of ultrasonic activation, the ultrasonic file or tip must be inserted passively inside the root canals to reduce the risk of fracture, cutting into the canal wall, and generating stronger acoustic streaming and cavitations. That is why the former concept of “active ultrasonic activation” has been replaced with “passive ultrasonic irrigation” [12]. Research has illustrated that the smaller the activated insert, the more powerful the cavitation and acoustic streaming that are produced within the solution, exerting greater shear stresses on the root canal walls and eliminating the apical vapour lock [6, 74]. Consequently, an ultrasonic tip of size 20, 0.01 taper was used in the current study and passively introduced up to 3 mm short of the working length [6, 74].

After completing the root canal preparation, adhesives were promptly applied to the dentine in the pulp chamber. This was done following a water rinse of the pulp chamber and gentle air drying to eliminate chemical residues and prevent excessive desiccation of the dentine. Next, sandblasting was performed using silica-coated alumina particles, which can enhance bond strength by improving the surface characteristics of dentine for both mechanical and chemical bonding. The treated surfaces were then rinsed with water and carefully air-dried to remove any residual debris created during the sandblasting process, thereby minimizing any negative impact on bond strength stability [86]. The immediate bonding process is essential in mitigating the potential adverse effects posed by residual irrigants, which may interfere with the integrity of the bonding interface. Furthermore, this procedure protects the dentine substrate against the different environmental factors that could jeopardize the coronal seal and subsequently the long-term treatment outcomes. By establishing a strong bonding interface, the risk of these effects is notably reduced, thereby improving the overall durability and effectiveness of the root canal treatment.

Universal adhesives could be classified into three main modes [87]; selective enamel etching, etch-and-rinse, and self-etch. Selective enamel etching focuses on etching the enamel only while utilizing the self-etching characteristics of the adhesive on dentine. The etch-and-rinse approach relies on pre-etching with phosphoric acid to achieve a robust bond, particularly to the enamel. The self-etch mode possesses numerous advantages such as being less technique-sensitive and allowing for simpler application since it incorporates etching and priming into a single step. Owing to avoiding the use of phosphoric acid which could desiccate collagen fibrils, this mode is typically associated with reduced postoperative sensitivity. Additionally, it permits superior monomer penetration into the dentine, thereby enhancing bond longevity and long-term success. Although selective enamel etching and etch-and-rinse approaches can provide high bond strength, the self-etch method strikes a balance between clinical practicality and bond quality, making it an appropriate option for various clinical scenarios. As a result, the self-etch method was chosen in the present study.

To establish accurate and reproducible micro-tensile bond strength values, it is essential to standardize the preparation of the beams employed in testing. The present study carefully selected the same anatomical areas for beam preparation across all samples, focusing on the axial buccal wall of the pulp chamber. This choice was based on prior research that underscored the suitability of this region for assessing the micro-tensile bond strength [88, 89]. The axial buccal wall offers a consistent bonding surface, reducing the variability associated with different dentine structures that might be encountered in other areas of the tooth. By using this specific region, we aimed to enhance the reliability of our results, ensuring that any variations in bond strength measurements can be attributed more confidently to the materials or techniques being tested rather than to disparities in sample preparation. This methodological method not only strived to present clearer insights into bond strength performance but also supported the generation of data that can be consistently replicated in future studies.

The conflicting results in the literature regarding the influence of the cross-head speed on the micro-tensile bond strength values, irrespective of the adhesive type [90–92], suggest a degree of strength in the testing methodology, allowing for more flexibility in experimental design. Accordingly, testing speeds of 0.5 mm/min and those of 1 mm/min are frequently adopted. Although the speed of 1 mm/min seems slightly faster, it remains within the range that does not compromise the bond strength results. Owing to its ability to offer well-controlled testing conditions and reduce the risk of introducing variables that may arise from faster testing speeds, facilitating the acquisition of accurate measurements of the micro-tensile bond strength [93], a cross-head speed of 0.5 mm was applied in the present study.

In this investigation, passive intermittent ultrasonic activation was compared to conventional syringe irrigation using the most commonly used irrigants. The study specifically focused on comparing irrigation techniques rather than assessing the effect of different irrigating solutions. As a result, non-irrigated samples or samples irrigated with saline or distilled water were not included.

Despite the reported beneficial role ultrasonic irrigation plays in root canal cleaning and disinfection [6, 15, 19], the present results raise concerns about its impact on the bond strength of composite resin coronal restoration. Therefore, the current study suggests immediate sealing of dentine before undergoing root canal treatment. Recent findings indicate that early dentine hybridization prior to root canal therapy considerably improves the micro-tensile bond strength between composite resin restorations and coronal dentine [94]. This enhancement reduces the need for re-preparation of the exposed dentine after root canal treatment, thus helping to preserve sound tooth structure [94].

If immediate pre-endodontic sealing is not undertaken, it becomes essential to treat the coronal dentine before placing the final adhesive restoration following root canal therapy. The application of a reducing agent, such as sodium ascorbate, followed by thorough rinsing with water, is particularly important after ultrasonic NaOCl irrigation. Sodium ascorbate effectively reacts with the oxygen by-products generated from NaOCl through a redox reaction. It has been shown that 10% sodium ascorbate applied for 2 min is able to notably enhance the bond strength to NaOCl-treated dentine [49, 95]. In addition, the present study suggests using the two-step self-etch adhesive system in root canal-treated teeth, particularly when ultrasonic irrigation is performed.

One of the limitations of the present study is its ex vivo design. However, ex vivo testing of materials, devices, and techniques under controlled conditions is essential as long as it provides a meaningful clinical translation. Modern scientific research focuses on enhancing the quality and accuracy of laboratory studies by developing guidelines that minimize bias resulting from academic freedom. This ensures precise study design, thorough implementation, and improved reporting, ultimately enhancing the clinical relevance of the outcomes. One of the strengths of the current laboratory study is its adherence to the Preferred Reporting Items for Laboratory Studies in Endodontics (PRILE 2021).

In laboratory studies aiming to test the effect of root canal irrigation on the micro-tensile bond strength of coronal restorations, it is important not to introduce other variables, such as sealer contamination and cleaning of the pulp chamber from residue of root filling material, between root canal irrigation and the application of coronal restorations [32, 34]. However, another limitation of the current experiment was that the root canal filling procedures were not carried out, which may diverge from the clinical situation. In order to foster the outcomes of the present experiment, future prospective randomized clinical trials with an adequate sample size are required to evaluate the durability and structural integrity of the coronal restoration following ultrasonic irrigation. 

## Conclusion

Within the constraints of the current laboratory investigation, ultrasonic irrigation adversely affected the strength of the bond between the composite resin coronal restorations and the pulp chamber dentine compared to conventional syringe irrigation. The two-step adhesive system was able to optimize bonding to the pulp chamber dentine when compared to the universal adhesive.

## Electronic supplementary material

Below is the link to the electronic supplementary material.

Supplementary Material 1: Supplementary Figure 1. PRILE 2021 flowchart.

Supplementary Material 2: Checklist of items to be included when reporting laboratory studies in Endodontology.

## Data Availability

All data or materials generated or analyzed during this study are included in this article.
