# Supplementary material for: The impact of passive ultrasonic irrigation on the bond strength of two different self-etch adhesives to human pulp chamber dentine: a laboratory investigation
Source: BMC Oral Health. 2025 Apr 11;25:550. doi: 10.1186/s12903-025-05858-x (PMC11992744; doi:10.1186/s12903-025-05858-x)
Supplement: Supplementary file 2 — Supplementary Material 2: Checklist of items to be included when reporting laboratory studies in Endodontology. [file 12903_2025_5858_MOESM2_ESM.docx]

**PRILE 2021**

**Checklist of items to be included when reporting laboratory studies in Endodontology***

| **Section/**  **Topic** | **Item Number** | **Checklist Items** | **Reported on page number** |
| --- | --- | --- | --- |
| **Title** | 1a | The Title must identify the study as being laboratory-based, e.g. “laboratory investigation”, “*in vitro*,” “*ex vivo*”, or another appropriate term | Title Page |
|  | 1b | The area/field of interest must be provided (briefly) in the Title | Title Page |
| **Keywords** | 2a | At least two keywords related to the subject and content of the investigation must be provided | Abstract Page |
| **Abstract** | 3a | The rationale/justification of what the investigation contributes to the literature and/or addresses a gap in knowledge must be provided | Abstract Page |
|  | 3b | The aim/objectives of the investigation must be provided | Abstract Page |
|  | 3c | The body of the Abstract must describe the materials and methods used in the investigation and include information on data management and statistical analysis | Abstract Page |
|  | 3d | The body of the Abstract must describe the most significant scientific results for all experimental and control groups | Abstract Page |
|  | 3e | The main conclusion(s) of the study must be provided | Abstract Page |
| **Introduction** | 4a | A background summary of the scientific investigation with relevant information must be provided | 2 |
|  | 4b | The aim(s), purpose(s), or hypothesis(es) of an investigation must be provided ensuring they align with the methods and results | 2 |
| **Materials and Methods** | 5a | A clear ethics statement and the ethical approval granted by an ethics board, such as an Institutional Review Board or Institutional Animal Care and Use Committee, must be described | 3 |
|  | 5b | When harvesting cells and tissues for research, all the legal, ethical, and welfare rights of human subjects and animal donors must be respected and applicable procedures described | Not applicable |
|  | 5c | The use of reference samples must be included, as well as negative and positive control samples, and the adequacy of the sample size justified | 5 |
|  | 5d | Sufficient information about the methods/materials/supplies/samples/specimens/instruments used in the study must be provided to enable it to be replicated | 3-7 |
|  | 5e | The use of categories must be defined, reliable, and be described in detail | 5-6 |
|  | 5f | The number of replicated identical samples must be described within each test group. The number of times each test was repeated must be described | 5-6 |
|  | 5g | The details of all the sterilization, disinfection, and handling conditions must be provided, if relevant | 3-7 |
|  | 5h | The process of randomization and allocation concealment, including who generated the random allocation sequence, who decided on which specimens to be included, and who assigned specimens to the intervention must be provided(if applicable) | 5 |
|  | 5i | The process of blinding the operator who is conducting the experiment (if applicable) and the examiners when assessing the results must be provided | Not applicable |
|  | 5j | Information on data management and analysis including the statistical tests and software used must be provided | 7 |
| **Results** | 6a | The estimated effect size and its precision for all the objectives (primary and secondary) for each group including controls must be provided | 7 |
|  | 6b | Information on the loss of samples during experimentation and the reasons must be provided, if relevant | Not relevant |
|  | 6c | All the statistical results, including all comparisons between groups, must be provided | 7 |
| **Discussion** | 7a | The relevant literature and status of the hypothesis must be described | 8 |
|  | 7b | The true significance of the investigation must be described | 7-9 |
|  | 7c | The strength(s) of the study must be described | 9 |
|  | 7d | The limitations of the study must be described | 9 |
|  | 7e | The implications for future research must be described | 9 |
| **Conclusion(s)** | 8a | The rationale for the conclusion(s) must be provided | 9 |
|  | 8b | Explicit conclusion(s) must be provided, i.e. the main “take-away” lessons | 9 |
| **Funding and support** | 9a | Sources of funding and other support (such as supply of drugs, and equipment) as well as the role of funders must be acknowledged and described | Not relevant |
| **Conflicts of interest** | 10a | An explicit statement on conflicts of interest must be provided | Title Page |
| **Quality of images** | 11a | Details of the relevant equipment, software, and settings used to acquire the image(s) must be described in the text or legend | Not applicable |
|  | 11b | If an image(s) is included in the manuscript, the reason why the image(s) was acquired and why it is included must be provided in the text | Not applicable |
|  | 11c | The circumstances (conditions) under which the image(s) were viewed and evaluated must be provided in the text | Not applicable |
|  | 11d | The resolution and any magnification of the image(s) or any modifications/ enhancements (e.g. brightness, image smoothing, staining, etc.) that were carried out must be described in the text or legend | Not applicable |
|  | 11e | An interpretation of the findings (meaning and implications) from the image (s) must be provided in the text | Not applicable |
|  | 11f | The legend associated with each image must describe clearly what the subject is and what specific feature(s) it illustrates | 12 |
|  | 11g | Markers/labels must be used to identify the key information in the image(s) and defined in the legend | Not applicable |
|  | 11h | If relevant, the legend of each image must include an explanation of whether it is pre-experiment, intra-experiment, or post-experiment and, if relevant, how images over time were standardized | 12 |

***From Nagendrababu V, Murray PE, Ordinola-Zapata R, Peters OA, Rôças IN, Siqueira JF Jr, Priya E, Jayaraman J, Pulikkotil SJ, Camilleri J, Boutsioukis C, Rossi-Fedele G, Dummer PMH (2021) PRILE 2021 guidelines for reporting laboratory studies in Endodontology: a consensus-based development. *International Endodontic Journal* May 3. doi: 10.1111/iej.13542.** [**https://onlinelibrary.wiley.com/doi/abs/10.1111/iej.13542**](https://onlinelibrary.wiley.com/doi/abs/10.1111/iej.13542)

**For further details visit: http://pride-endodonticguidelines.org/prile**
